# A computed tomography-based preoperative risk scoring system to distinguish lymphoepithelioma-like gastric carcinoma from non-lymphoepithelioma-like gastric carcinoma

**DOI:** 10.3389/fonc.2022.872814

**Published:** 2022-09-15

**Authors:** Liming Li, Wenpeng Huang, Ping Hou, Weiwei Li, Menyun Feng, Yiyang Liu, Jianbo Gao

**Affiliations:** ^1^ Department of Radiology, The First Affiliated Hospital of Zhengzhou University, Zhengzhou, China; ^2^ Department of Gastrointestinal Tract, Henan Key Laboratory of Imaging Diagnosis and Treatment for Digestive System Tumor, Henan, China

**Keywords:** gastric cancer, lymphoepithelioma-like carcinoma, tomography, X-ray computed, Epstein-Barr virus, nomogram

## Abstract

**Purpose:**

The aim of this study was to develop a preoperative risk scoring model for distinguishing lymphoepithelioma-like gastric carcinoma (LELGC) from non-LELGC based on contrast-enhanced computed tomography (CT) images.

**Methods:**

Clinicopathological features and CT findings of patients with LELGC and non-LELGC in our hospital from January 2016 to July 2022 were retrospectively analyzed and compared. A preoperative risk stratification model and a risk scoring system were developed using logistic regression.

**Results:**

Twenty patients with LELGC and 40 patients with non-LELGC were included in the training cohort. Significant differences were observed in Epstein–Barr virus (EBV) infection and vascular invasion between the two groups (*p* < 0.05). Significant differences were observed in the distribution of location, enhancement pattern, homogeneous enhancement, CT-defined lymph node status, and attenuations in the non-contrast, arterial, and venous phases (all *p* < 0.05). Enhancement pattern, CT-defined lymph node status, and attenuation in venous phase were independent predictors of LELGC. The optimal cutoff score of distinguishing LELGC from non-LELGC was 3.5. The area under the receiver operating characteristic curve, sensitivity, specificity, and accuracy of risk identification model in the training cohort were 0.904, 87.5%, 80.0%, and 85.0%, respectively. The area under the receiver operating characteristic curve, sensitivity, specificity, and accuracy of risk identification model in the validation cohort were 0.705 (95% CI 0.434–0.957), 75.0%, 63.6%, and 66.7%, respectively.

**Conclusion:**

A preoperative risk identification model based on CT imaging data could be helpful for distinguishing LELGC from non-LELGC.

## Introduction

Lymphoepithelioma-like gastric carcinoma (LELGC) is a distinct subtype of gastric cancer and accounts for approximately 1%–4% of all gastric cancers (1). LELGC exhibits characteristic histological features. Watanabe et al. first described this lymphocyte-rich cancer. Burke et al. first explained that this cancer had a close association with Epstein–Barr virus (EBV) (2, 3). Another pathological type of LELGC is microsatellite instability (MSI)-high carcinomas (1, 4). Due to its special pathological characteristics, LELGC has a better prognosis than non-LELGC (5, 6). Preoperative diagnosis facilitates the individualized treatment of LELGC patients (7). The endoscopic local treatment can be considered for LELGC in the early stage, because few tumors showed metastatic lymph nodes (6). Interestingly, previous research considered that quercetin was a potential antitumor agent in mice injected with EBV human gastric carcinoma cells (8).

Preoperative biopsy is a common method of tumor diagnosis. However, it is an invasive examination and has limited specimens. Computed tomography (CT) is a commonly used examination for gastric tumors (9–12). To the best of our knowledge, most previous related studies focused only on clinicopathological characteristics and few studies focused on CT characteristics. A previous study (13) in our medical center analyzed the CT features of four patients with LELGC, without control group with non-LELGC. Thus, we compared CT characteristics between patients with LELGC and non-LELGC, aiming to develop a preoperative risk scoring system to distinguish them.

## Materials and methods

### Patients

The present study was approved by the institutional review board of the authors’ hospital. Given the retrospective design of the study and the use of anonymized patient data, the requirement for informed consent was waived. Patients with pathologically confirmed LELGC from January 2016 to January 2021 were identified from the hospital database, and those with complete clinical and CT data were included. Individuals with undetectable lesions on CT imaging were excluded. A total of 20 patients fulfilled the inclusion criteria. At the same time, 40 non-LELGC patients with the same T stage distribution were identified from January 2021 to July 2021. In addition, a validation cohort was identified including 4 LELGC patients from February 2021 to July 2022 and 11 non-LELGC patients from June 2021 to July 2022.

### CT image acquisition

Patients were asked to ingest 600–1,000 ml of water before undergoing CT. CT images were captured on two scanners (Discovery CT750 HD, GE Healthcare, Milwaukee, USA; Brilliance iCT, Philips Healthcare, Best, the Netherlands). Scanning parameters were as follows: tube voltage, 120 kVp; tube current, 100–650 mAs; slice thickness and increments, 5 mm. Iodinated contrast agent (70–100 ml of 370 mg I/ml) was injected at a rate of 3 ml/s. Unenhanced phase and two enhanced phase images were obtained. Image acquisition of arterial and venous phases began at 10 s and 50–65 s after the attenuation of abdominal aorta reached 120 Hounsfield units (HU), using a bolus-tracking technique.

### Clinical and image analysis

An experienced radiologist reviewed clinical and pathological data. Clinical data included age and sex; meanwhile, pathological data included vascular invasion, perineuronal invasion, EBV infection, and Ki-67 index.

CT features were analyzed by two experienced radiologists blinded to the pathological results until a consensus is reached. CT features included location, enhancement pattern (gradual, continuous, and washed out), enhancement grade (mild, moderate, and obvious), homogeneous enhancement, ulcer appearance, circum wall involvement, and CT-defined lymph node status. The enhancement grade was evaluated based on CT attenuation (13). Thickness of tumor and CT attenuations at the non-contrast, arterial, and venous phases (CT_non_, CT_artery_, and CT_venous_) were measured. A region of interest was placed at the center of the mass, ranging from 9 mm^2^ to 60 mm^2^. All measurements were repeated three times at the maximum axial section, and mean values were used for analysis. A lymph node with an obvious enhancement or a short diameter greater than 8 mm was considered to be a CT-defined pathological lymph node (14, 15). A lymph node was classified as obvious enhancement with an attenuation of more than 40 HU.

### Statistical analysis

All statistical calculations were performed using SPSS version 22.0 (IBM Corporation, Armonk, NY, USA), MedCalc version 15.2 (MedCalc, Ostend, Belgium) and R software (version 3.5.0; http://www.Rproject.org).

Categorical variables (sex, vascular invasion, perineuronal invasion, EBV infection, location, enhancement pattern, enhancement grade, homogeneous enhancement, ulcer appearance, and circum wall involvement) were expressed as frequencies and were compared between groups using the Pearson *χ* ^2^ test or Fisher’s test. Continuous variables were expressed as mean and standard deviation (normal distribution) or median and interquartile range (non-normal distribution) and were compared between groups using independent sample *t*-test or Mann–Whitney *U* test. Variables with *p* < 0.05 were included in multivariate logistic regression analysis. Receiver operating characteristic (ROC) curves of variables were plotted and optimal cutoff values were generated. The areas under ROC curve (AUCs) were calculated and compared between different variables using the DeLong test. Continuous data were transformed into classified data according to cutoff values for subsequent analysis. A logistic regression model was built and risk factors were assigned scores according to the method used in the Framingham study (16, 17).

## Results

### Clinical analysis

Sixty patients were included in the training cohort, with a T-stage distribution as follows: T1 (5%), T2 (25%), T3 (35%), and T4 (35%). There are 3 (8%) signet ring cell carcinomas, 1 (2%) mucinous adenocarcinoma, and 36 (90%) tubular or papillary adenocarcinomas in the non-LELGC group. There were 18 (45%) poorly differentiated carcinomas, 21 (53%) moderately differentiated carcinomas, and 1 (2%) well-differentiated carcinoma in the non-LELGC group. Fifteen patients were included in the validation cohort with 4 LELGC and 11 non-LELGC patients. There are 1 (9%) signet ring cell carcinoma and 10 (91%) tubular or papillary adenocarcinomas in the non-LELGC group. There were 5 (45%) poorly differentiated carcinomas and 6 (55%) moderately differentiated carcinomas.

Clinical and pathological characteristics are summarized in **Table 1**. In the training cohort, there was no significant difference in terms of age, sex, perineuronal invasion, and Ki-67 index (all *p* > 0.05); EBV infection and vascular invasion differed significantly between two groups (*p* = 0.001 and 0.010, respectively).

**Table 1:** Univariate analysis of clinicopathological characteristics in patients.

Characteristics	Variable	Training Cohort			Validation Cohort		*p*-value
		LELGC (*n* = 20) (%)	Non-LELGC (*n* = 40) (%)	*p*-value	LELGC (*n* = 4) (%)	Non-LELGC (*n* = 11) (%)	
Age (years)		56 ± 10.97	60.2 ± 9.18	0.121	54.25 ± 4.92	60.82 ± 7.62	0.780
Sex	Male	16 (80)	31 (78)	1.000	3 (75)	10 (91)	0.476
	Female	4 (20)	9 (22)		1 (25)	1 (9)	
EBV	Positive	20 (100)	0 (0)	0.001^*^	4 (100)	0 (0)	0.001^*^
	Negative	0 (0)	40 (100)		0 (0)	11 (100)	
Perineuronal invasion	Present	13 (65)	30 (75)	0.418	2 (50)	4 (36.4)	0.538
	Absent	7 (35)	10 (25)		2 (50)	7 (63.6)	
Vascular invasion	Present	7 (35)	28 (70)	0.010^*^	2 (50)	5 (45.5)	0.662
	Absent	13 (65)	12 (30)		2 (50)	6 (54.5)	
Ki-67 index		70 (52.5, 80.0)	60 (40, 70.0)	0.077	65 (45.0, 77.5)	70 (50.0, 80.0)	1.000
T stage	1	1 (5)	2 (5)	1.000	1 (25)	0 (0)	0.275
	2	5 (25)	10 (25)		1 (25)	2 (18.2)	
	3	7 (35)	14 (35)		2 (50)	7 (63.6)	
	4	7 (35)	14 (35)		0 (0)	2 (18.1)	

EBV, Epstein–Barr virus; ^*^p-value < 0.05.

### CT image analysis

**Figure 1:**
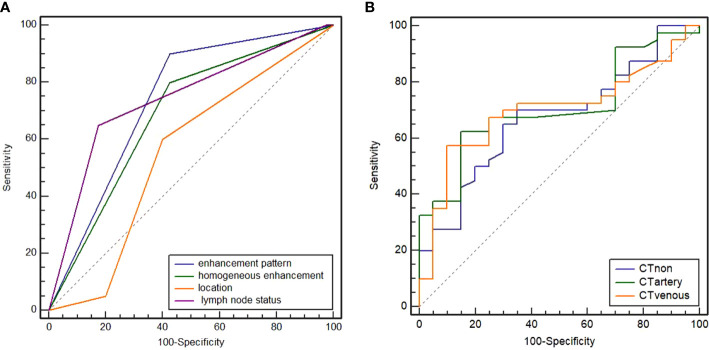
Receiver operating characteristic (ROC) curves of enhancement pattern, homogeneous enhancement, CT-defined lymph node status, and location **(A)**. The areas under curve (AUC) were 0.716, 0.645, 0.738, and 0.543, respectively. ROC curves of attenuation in non-contrast, arterial, and venous phases **(B)**. The AUC were 0.673, 0.709, and 0.699, respectively.

**Figure 2:**
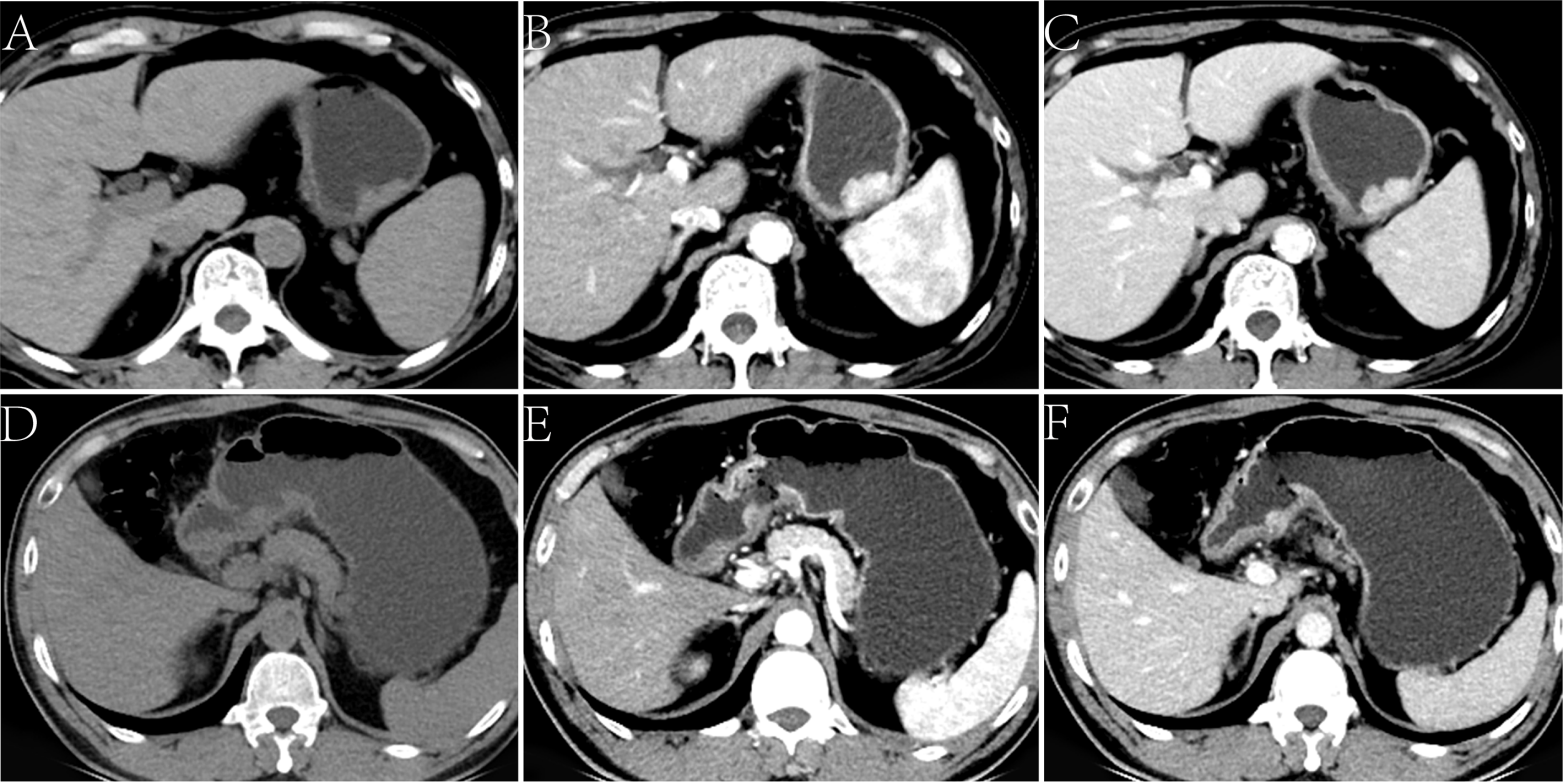
Lymphoepithelioma-like gastric carcinoma (LELGC) in a 60-year-old man **(A-C)** and non-LELGC in a 51-year-old woman **(D-F)**. Unenhanced CT image of stomach reveals a mass in the gastric body **(A)**. Contrast-enhanced CT image shows a continuously obvious homogeneous enhancement of mass **(B, C)** with CT_venous_ of 110.20 HU. Unenhanced CT image of stomach reveals a mass in the gastric antrum **(D)**. Contrast-enhanced CT image shows a gradually moderate nonhomogeneous enhancement of mass **(E, F)** with a CT_venous_ of 67.16 HU.

The CT findings of the two groups are summarized in **Table 2**. In the training cohort, significant differences were observed in the distribution of location, enhancement pattern, homogeneous enhancement, CT-defined lymph node status, CT_non_, CT_artery_, and CT_venous_ (all *p* < 0.05). The AUCs of the above seven variables were 0.543 (95% confidence interval [CI] 0.376–0.710), 0.716 (95% CI 0.568–0.864), 0.645 (95% CI 0.478–0.811), 0.738 (95% CI 0.577–0.900), 0.673 (95% CI 0.539–0.788), 0.709 (95% CI 0.578–0.819), and 0.699 (95% CI 0.567–0.810), respectively (**Figure 1**). Most LELGCs were located in gastric cardia, fundus, and body, with homogeneous and continuous enhancement (**Figure 2**).

**Table 2:** Univariate analysis of CT characteristics in patients.

		Training Cohort			Validation Cohort		*p*-value
Characteristics	Variable	LELGC (*n* = 20) (%)	Non-LELGC (*n* = 40) (%)	*p*-value	LELGC (*n* = 4) (%)	Non-LELGC (*n* = 11) (%)	
Location	Cardia, fundus	8 (40)	24 (60)	0.017^*^	1 (25)	3 (27)	0.174
	Body	11 (55)	8 (20)		3 (75)	3 (27)	
	Antrum	1 (5)	8 (20)		0 (0)	5 (45)	
Ulcer	Present	14 (70)	30 (75)	0.680	2 (50)	5 (45)	0.662
	Absent	6 (30)	10 (25)		2 (50)	6 (55)	
Enhancement pattern	Continuously	18 (90)	17 (43)	0.001^*a^	2 (50)	3 (27)	0.407
	Gradually	2 (10)	21 (52)		2 (50)	8 (73)	
	Washed out	0 (0)	2 (5)		0 (0)	0 (0)	
Enhancement grade	Obvious	10 (50)	20 (50)	1.000^a^	2 (50)	6 (55)	0.958
	Moderate	9 (45)	16 (40)		1 (25)	3 (27)	
	Mild	1 (5)	4 (10)		1 (25)	2 (18)	
Homogeneous enhancement	Present	16 (80)	17 (43)	0.006^*^	3 (75)	4 (36)	0.231
	Absent	4 (20)	23 (57)		1 (25)	7 (64)	
Circum wall involvement	Present	3 (15)	10 (25)	0.580	0 (0)	3 (27)	0.363
	Absent	17 (85)	30 (75)		4 (100)	8 (73)	
CT-defined lymph node status	Unnormal	7 (35)	32 (80)	0.001^*^	2 (50)	7 (64)	0.538
	Normal	13 (65)	8 (20)		2 (50)	4 (36)	
Thickness (mm)		11.5 (2.85, 21.00)	14.27 (11.50, 17.23)	0.371	16.50 (10.66, 25.71)	15.20 (10.59, 25.17)	0.694
CT_non_ (HU)		42.35 ± 6.47	37.78 ± 7.63	0.022^*^	36.50 ± 10.79	36.55 ± 9.54	0.994
CT_artery_ (HU)		87.00 ± 15.41	70.59 ± 21.21	0.003^*^	65.50 (37.93, 110.10)	62.30 (42.67, 102.50)	1.000
CT_venous_ (HU)		91.57 ± 14.66	79.71 ± 19.79	0.021^*^	89.50 (44.00, 124.50)	78.00 (56.80, 102.40)	0.948

CT_non_, CT_artery_, and CT_venous_: the attenuations in non-contrast, arterial, and venous phase, respectively. ^*^p-value < 0.05. ^a^Merging data of adjacent rows appeared.

In the training cohort, there was no remarkable difference in AUC between different phase attenuations (all *p* > 0.05), and also no remarkable difference in AUC between location, enhancement pattern, homogeneous enhancement, and CT-defined lymph node status (all *p* > 0.05). The cutoff values determined by the ROC curve for CT_non_, CT_artery_, and CT_venous_ were 41.84 HU, 83.00 HU, and 82.73 HU, respectively. No statistical difference was observed in ulcer appearance, circum wall involvement, enhancement grade, and thickness between two groups (*p* > 0.05).

### Logistic regression analysis

**Figure 3:**
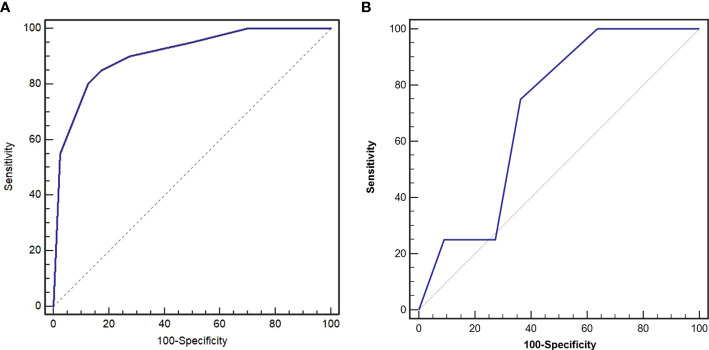
Receiver operating characteristic (ROC) curves of preoperative risk identification model in the training cohort **(A)** and validation cohort **(B)**. The area under the curve (AUC) was 0.904 and 0.705, respectively.

On logistic regression analysis, the odds ratios (ORs) remained significant for attenuation in the venous phase (*p* = 0.002), CT-defined lymph node status (*p* = 0.039), and enhancement pattern (*p* = 0.005). The Hosmer–Lemeshow test revealed that the model had a good fit (*p* = 0.986). The AUC of the model in the training data set was 0.904 (95% CI 0.803–0.966), and the sensitivity, specificity, and accuracy were 87.5%, 80.0%, and 85.0%, respectively (**Table 3**). The AUC of the model in the validation cohort was 0.705 (95% CI 0.434–0.957), and the sensitivity, specificity, and accuracy were 75.0%, 63.6%, and 66.7%, respectively (**Figure 3**).

**Table 3:** Multivariate analysis in identifying LELGC from non-LELGC.

Parameter	*β*	Odds ratio	95% confidence interval		*p*-value
			Lower	Upper	
Constant	−7.877				0.001
Enhancement pattern	2.761	15.812	2.440	102.450	0.004
CT-defined lymph node status	1.657	5.245	1.074	25.610	0.041
CT_venous_	3.053	21.175	3.1546	142.510	0.002

CT_venous_: the attenuations in venous phase.

### Development of a preoperative risk stratification system

**Figure 4:**
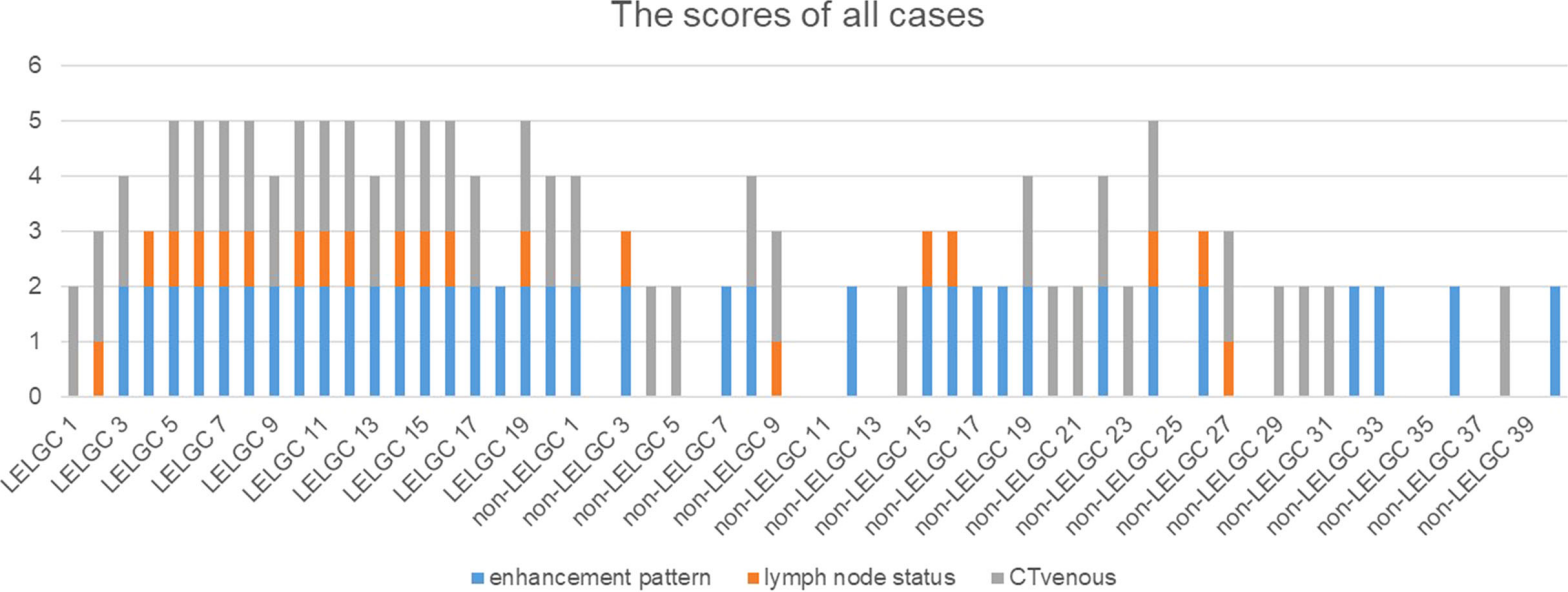
The preoperative risk scores of patients in the training cohort. The total scores of patients with lymphoepithelioma-like gastric carcinoma (LELGC) were higher than that of patients with non-LELGC.

**Figure 5:**
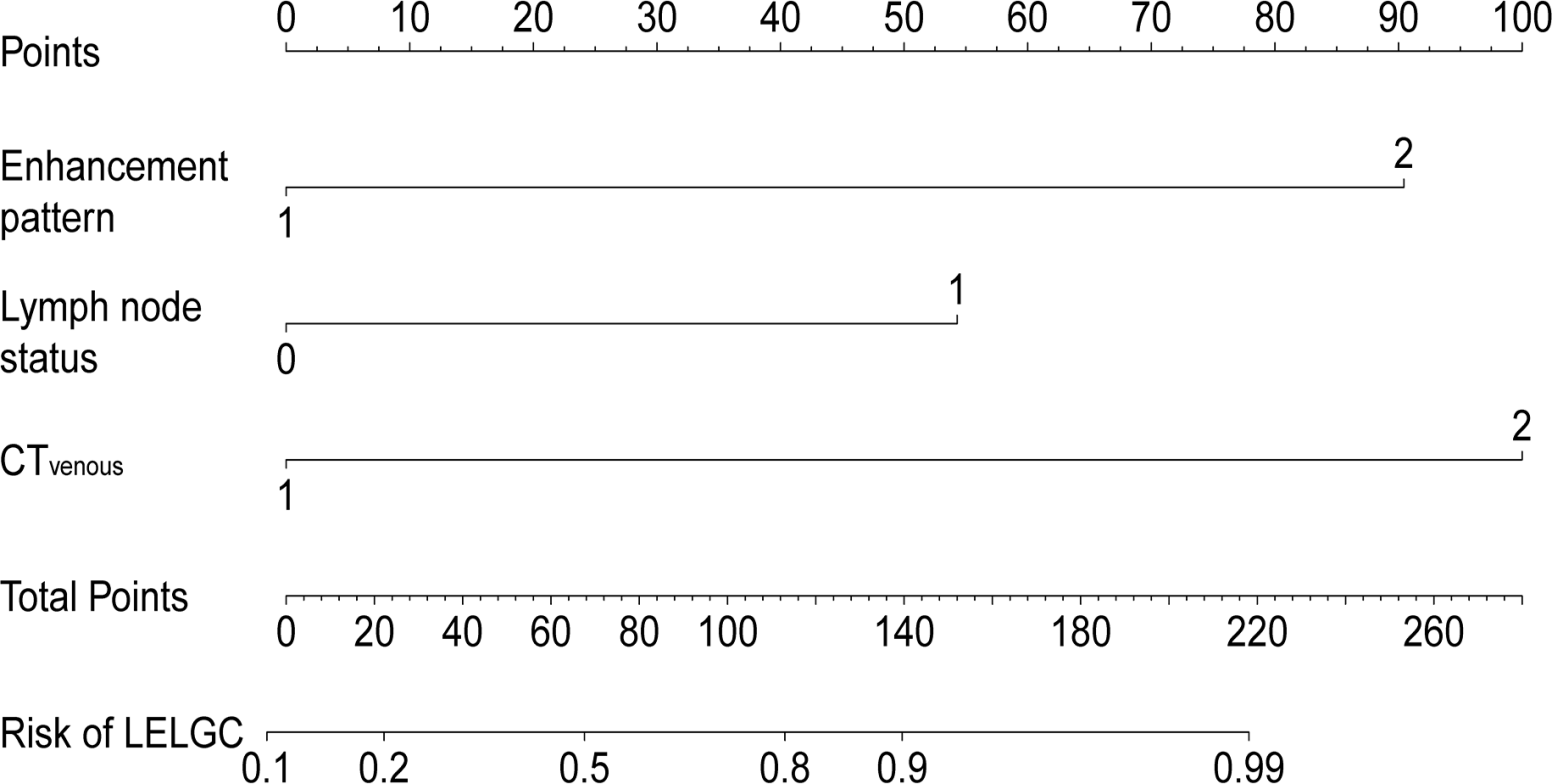
Nomogram for predicting lymphoepithelioma-like gastric carcinoma (LELGC). The CT_venous_, CT-defined lymph node status, and enhancement pattern were incorporated into nomogram finally.

A preoperative risk stratification model was developed based on three risk factors. The total scores of risk factors in the training cohort are shown in **Figure 4** and **Table 4**. The nomogram for predicting LELGC is shown in **Figure 5**. The optimal cutoff score was 3.5.

**Table 4:** The score for preoperative risk factors.

Variable	Regression coefficient	Category	Score
Enhancement pattern	2.761		
		Continuously	2
		Other	0
CT-defined lymph node status	1.657		
		Normal	1
		Unnormal	0
CT_venous_	3.053		
		>82.73	2
		≤82.73	0

CT_venous_: the attenuations in venous phase.

## Discussion

It is important to preoperatively distinguish LELGC from non-LELGC with the increasing application of neoadjuvant therapy. We developed a CT-based risk scoring system to preoperatively distinguish LELGC from non-LELGC. The system incorporated three variables: enhancement pattern, CT-defined lymph node status, and CT_venous_. When risk score is greater than 3.5, the tumor was more inclined to be a LELGC.

LELGC is common in the elderly population, predominantly in men, similar to non-LELGC. Men were more prone to be observed in the LELGC than in the non-LELGC group in a previous study (2). The results of the present study revealed that the LELGC group exhibited less vascular invasion than the non-LELGC group. Vascular invasion is an independent risk factor for poor survival in GC after curative resection (18). Less vascular invasion represents a relatively favorable prognosis of LELGC.

Lately, EBV-positive gastric cancer has attracted wide attention after the new classification proposed by The Cancer Genome Atlas research. More than 80% of LELGC cases are EBV-positive and approximately 10% of LELGC cases exhibit MSI (4, 19, 20). The presence of high-frequency MSI appeared in EBV-negative LELGC cases, which suggested that EBV and MSI had different pathogenic patterns for LELGC (21). A previous study showed that EBV infection was an independent predictor for survival in LELGC patients (22). All LELGCs in our study were EBV-positive, and no patients exhibited MSI. This may be explained by the late detection of MSI in our hospital.

There is a significant difference in the distribution of location between two groups. The majority of LELGCs are located in the gastric cardia, fundus, and body, and few tumors are located in the gastric antrum, which is consistent with previous studies. In addition, LELGC in the early T stages also needs to be differentiated from submucosal tumors (23).

A small number of LELGC tumors had metastatic lymph nodes, which was consistent with the previous study (4). In practice, there are no universally accepted criteria to define pathological lymph nodes at CT images. However, CT-defined lymph node status, like the deep learning radiomics characteristics, was included in the final nomogram to predict lymph node metastasis and exhibited significant predictive value in previous studies (10).

It was interesting to find that enhancement pattern was highly significant in distinguishing two tumors. Homogeneous and continuous enhancement was more often observed in the LELGC group than in the non-LELGC group. This seems to conflict with previous research. It is generally accepted that most of the undifferentiated types of gastric cancers have a peak enhancement in delayed phase (24). The CT enhancement is closely influenced by pathological components. The histological basis of gradual enhancement is mainly caused by fibrous tissue (25). LELGC is a poorly differentiated tumor with a large number of lymphocytes infiltrating the stroma and exhibits early enhancement in arterial phase and homogeneous enhancement in venous phase. However, most advanced gastric cancers exhibit gradual enhancement with abundant fibrous connective tissue and a small amount of scattered lymphocyte infiltrating the stroma (26).

There were also some limitations that warrant discussion. First, we selected the control non-LELGC group from the nearest time according to T stage, which inevitably introduced bias. Second, because the LELGC was rare and the study was about the CT characteristics, the cohort of patients is small. A larger sample size comprising multicenter data is needed to verify our risk scoring system. The third crucial point was the nature of retrospective analysis; the impacts of the heterogeneities of CT scanners on the attenuation were not explicitly considered.

## Conclusion

In conclusion, a preoperative risk scoring system based on enhancement pattern, CT-defined lymph node status, and attenuation in venous phase was effective in distinguishing LELGC from non-LELGC. This system could be helpful for individualized treatment strategies for patients with LELGC.

## Data availability statement

The raw data supporting the conclusions of this article will be made available by the authors, without undue reservation.

## Ethics statement

This study was reviewed and approved by the Medical Ethics Committee of Zhengzhou University. The patients/participants provided their written informed consent to participate in this study. Written informed consent was obtained from the individual(s) for the publication of any potentially identifiable images or data included in this article.

## Author contributions

LL: manuscript preparation, and data collection and analysis. WH: literature research and data analysis. YM: guidance of pathological knowledge. PH: guidance of imaging knowledge. WL: imaging data collection and analysis. MF: manuscript editing. JG: study conception and design, manuscript review, and guarantor of integrity of the entire study. All authors contributed to the article and approved the submitted version.

## Funding

This study has received funding by National Natural Science Foundation of China (Grant No. 81971615).

## Conflict of interest

The authors declare that the research was conducted in the absence of any commercial or financial relationships that could be construed as a potential conflict of interest.

## Publisher’s note

All claims expressed in this article are solely those of the authors and do not necessarily represent those of their affiliated organizations, or those of the publisher, the editors and the reviewers. Any product that may be evaluated in this article, or claim that may be made by its manufacturer, is not guaranteed or endorsed by the publisher.
